# Evolving Global Burden of GBD-Defined Spinal Cord Lesions, 2014–2023: A Global Burden of Disease Analysis of the Geriatric Transition and Anatomical Distribution

**DOI:** 10.3390/jcm15145672

**Published:** 2026-07-20

**Authors:** Negin Fani, Jerzy Andrzej Gregorczyk, Wojciech Bajda, Mikołaj Biegański, Kajetan Latka, Piotr Dąbrowski, Julia Nosko, Mateusz Bielecki

**Affiliations:** 1 Medical University of Warsaw, 02-091 Warsaw, Poland; 2 Department of Neurology, St. Hedwig’s Regional Specialist Hospital, Institute of Medical Sciences, University of Opole, Wodociagowa 4, 45-221 Opole, Poland; 3 Medical Faculty, Lazarski University in Warsaw, 02-662 Warsaw, Poland; 4 Neurosurgery, John Paul II Western Hospital in Grodzisk Mazowiecki, 05-825 Grodzisk Mazowiecki, Poland

**Keywords:** GBD-defined spinal cord lesion, spinal cord injury, Global Burden of Disease, epidemiology, disability, years lived with disability, aging, Socio-demographic Index, cervical lesion, rehabilitation

## Abstract

**Background**: Global Burden of Disease (GBD)-defined spinal cord lesion categories are major contributors to global disability. However, contemporary analyses focusing on age-specific disability burden, socio-demographic variation, and anatomical distribution remain limited. This study aimed to analyze global and regional trends in prevalence and disability burden associated with GBD-defined spinal cord lesions from 2014 to 2023, with particular emphasis on working-age adults aged 15–49 years, older adults aged ≥70 years, Socio-demographic Index (SDI) regions, and anatomical level. **Methods**: We performed a secondary analysis of Global Burden of Disease (GBD) 2023 Injuries by Nature data, extracting estimates for “spinal cord lesion at neck level” and “spinal cord lesion below neck level.” Global and regional prevalence and Years Lived with Disability (YLDs) were extracted. The cervical ratio was calculated as the proportion of total YLDs attributable to neck-level lesions. Temporal trends were assessed using log-linear regression and annual percent change (APC). **Results**: Between 2014 and 2023, the global age-standardized prevalence rate decreased from 363.17 to 318.28 per 100,000 population, corresponding to a 12.4% reduction (APC −1.97%/year; *p* = 0.002). The global age-standardized YLD rate decreased by 14.0% (APC −2.19%/year; *p* = 0.001). Absolute prevalence remained relatively stable, increasing slightly from 27.31 million to 27.54 million, while absolute YLDs changed from 8.16 million to 8.02 million without a significant linear trend. A clear age-related divergence was observed: YLDs increased by 31.5% among adults aged ≥70 years (APC +2.57%/year; *p* < 0.001), while they decreased by 11.7% among individuals aged 15–49 years (APC −1.87%/year; *p* = 0.005). Cervical-level lesions accounted for 63.5% of global YLDs in 2023, and the cervical ratio increased stepwise from low-SDI to high-SDI regions. **Conclusions**: Although age-standardized prevalence and YLD rates associated with GBD-defined spinal cord lesions declined globally, the absolute burden remained relatively stable and shifted toward older adults. These findings highlight the importance of considering age structure, socio-demographic development, and anatomical distribution when planning rehabilitation, long-term care, and health-system resources for populations affected by GBD-defined spinal cord lesions.

## 1. Introduction

Global Burden of Disease (GBD)-defined spinal cord lesions remain an important source of disability and healthcare burden worldwide, particularly among older adults [1]. Affected individuals may experience neurological deficits, reduced quality of life (QOL), reduced life expectancy, and substantial economic burden [2,3]. The epidemiology of spinal cord injury and related GBD-defined spinal cord lesion categories has changed over time, reflecting factors such as injury-prevention strategies, trauma systems, demographic shifts, and access to acute and rehabilitative care [4]. Understanding recent epidemiological patterns may help clinicians and policymakers anticipate changing needs in spine care, rehabilitation, and long-term disability services. Previous studies have demonstrated that the burden of spinal cord injury and related GBD-defined spinal cord lesion categories varies by geography, time period, and socio-demographic context [5,6]. Large-scale epidemiological analyses are therefore essential for characterizing these trends, as individual registries and hospital-based studies are often limited by geography, sample size, case definitions, or short follow-up periods.

The GBD database provides modeled estimates of disease and injury burden using standardized methods across time, age groups, sexes, and regions. In the GBD Injuries by Nature framework, spinal cord lesion categories are modeled separately as “spinal cord lesion at neck level” and “spinal cord lesion below neck level” [7]. Therefore, the present analysis does not capture all spinal trauma or all clinically adjudicated neurological spinal cord injuries but specifically evaluates the modeled burden of these two GBD-defined spinal cord lesion categories. For readability, these categories are referred to as GBD-defined spinal cord lesions throughout the manuscript.

This study aimed to analyze global and regional trends in prevalence and disability burden associated with GBD-defined spinal cord lesions from 2014 to 2023, with particular focus on differences between working-age adults (15–49 years) and older adults (≥70 years). We further examined disparities across Socio-demographic Index (SDI) regions and assessed the relative contribution of cervical versus below-neck lesion categories to the global YLD burden. By integrating age-, region-, and anatomical-level analyses, this study provides an updated perspective on the evolving global burden of GBD-defined spinal cord lesions and highlights implications for spine care planning, rehabilitation services, and health policy in aging societies.

## 2. Methods

### 2.1. Study Design and Data Source

This study is a secondary analysis of publicly available data from the Global Burden of Disease (GBD) 2023 Study [8], coordinated by the Institute for Health Metrics and Evaluation (IHME). The GBD study uses standardized and reproducible methods to generate modeled estimates of disease burden across countries, age groups, sexes, and time periods. All data used in this analysis are de-identified and freely accessible through the GBD Results Tool (VizHub 2023 Model).

### 2.2. Case Definition

Cases were identified using the GBD 2023 Injuries by Nature classification [8,9]. Specifically, we extracted estimates for “spinal cord lesion at neck level” and “spinal cord lesion below neck level.” In this manuscript, “GBD-defined spinal cord lesions” refers to the combined modeled burden of these two GBD Injuries by Nature categories. Because GBD estimates are modeled population-level injury categories rather than individual-level clinical diagnoses, these estimates should not be interpreted as exclusively representing clinically confirmed neurological spinal cord injury or all forms of spinal trauma. Anatomical injury level was classified as cervical/neck level versus below-neck level according to GBD definitions.

### 2.3. Measures

The primary outcomes of interest were prevalence and Years Lived with Disability (YLDs). For each outcome, both age-standardized rates per 100,000 population and absolute numbers were extracted when available. YLDs were used as the principal metric of disability burden, reflecting the chronic functional impairment associated with GBD-defined spinal cord lesions and their implications for long-term healthcare demand. Although incidence and disability-adjusted life years (DALYs) are available within the GBD framework, this analysis focused on prevalence and YLDs to better capture long-term disability patterns, particularly in aging populations.

### 2.4. Population and Stratification

Analyses were conducted globally and included both sexes. Age-specific analyses were broken down into two groups: Working-age adults (15–49 years) and the elderly (≥70 years). To assess the influence of socio-demographic development, results were further broken down by the Socio-demographic Index (SDI) quintiles: low, low-middle, middle, high-middle, and high SDI.

### 2.5. Anatomical Burden Assessment

We further evaluated anatomical injury level by calculating the cervical ratio, defined as the proportion of total GBD-defined spinal cord lesion-related YLDs attributable to neck-level lesions. The analysis investigated how this proportion changed over time and varied across SDI regions.

### 2.6. Statistical Analysis

Temporal trends from 2014 to 2023 were evaluated using annual GBD estimates. For each outcome, relative percentage change was first calculated between 2014 and 2023. To assess temporal trends across the full study period, log-linear regression models were fitted using the natural logarithm of the annual estimate as the dependent variable and calendar year as the independent variable. Annual percent change was calculated as APC = [exp(β) − 1] × 100, where β represents the regression coefficient for calendar year. Ninety-five percent confidence intervals and *p*-values were derived from the regression model. Analyses were performed for global burden metrics, age-specific YLDs and prevalence, SDI-stratified outcomes, and anatomical injury-level categories. Between-group comparisons were interpreted descriptively unless directly supported by trend estimates. Statistical analyses were performed using Python version 3.14.6 and verified manually against the original GBD 2023 data.

### 2.7. Ethics Statement

This study utilized publicly available, de-identified data and did not require Institutional Review Board approval or informed consent. AI-assisted tools were used to support language editing and figure generation. All numerical results, statistical analyses, figure data, and manuscript conclusions were verified by the authors against the original GBD 2023 dataset. AI-assisted tools were not used to generate or interpret statistical results, make scientific conclusions, or replace author judgment.

## 3. Results

**Figure 1:**
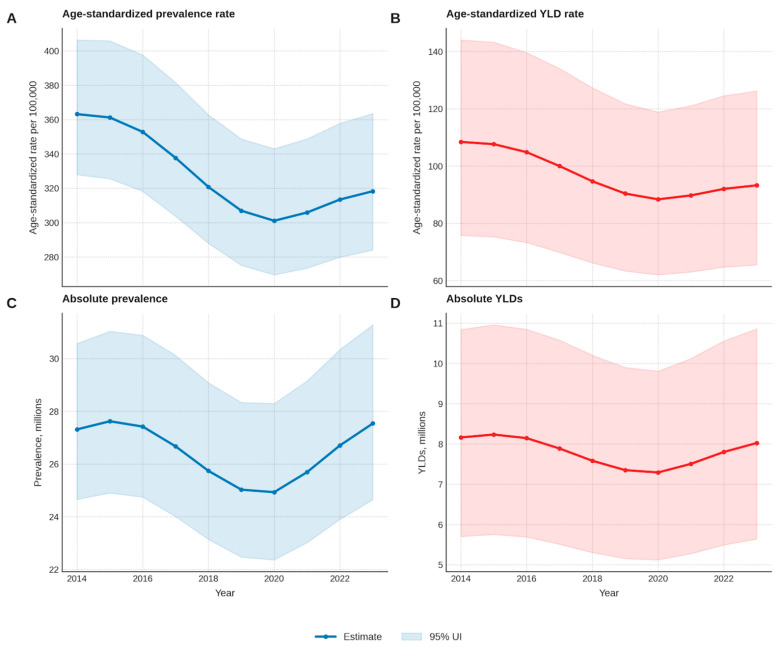
Global trends in **GBD-defined spinal cord lesion burden**, 2014–2023. (**A**) Age-standardized prevalence rate per 100,000 population. (**B**) Age-standardized YLD rate per 100,000 population. (**C**) Absolute prevalence in millions. (**D**) Absolute YLDs in millions. Estimates represent the summed burden of spinal cord lesion at neck level and spinal cord lesion below neck level from the GBD 2023 Injuries by Nature dataset. Lines represent specific data points. Shaded bands (red and blue areas) represent 95% uncertainty intervals.

The global burden of GBD-defined spinal cord lesions exhibited a divergence between age-standardized rates and absolute numbers over the last decade (Figure 1A–D). Between 2014 and 2023, the global age-standardized prevalence rate decreased from 363.17 (95% UI: 327.87–406.32) to 318.28 (95% UI: 284.00–363.52) per 100,000 population, corresponding to a 12.4% reduction (Figure 1A). Log-linear trend analysis confirmed a significant annual decline in age-standardized prevalence rate over the study period (APC −1.97%/year; 95% CI: −2.98 to −0.95; *p* = 0.002). Similarly, the global age-standardized YLD rate decreased from 108.41 (95% UI: 75.76–144.00) to 93.25 (95% UI: 65.44–126.19) per 100,000 population, corresponding to a 14.0% reduction (Figure 1B). This decline was also statistically significant on log-linear trend analysis (APC −2.19%/year; 95% CI: −3.23 to −1.13; *p* = 0.001).

In contrast, absolute global burden remained relatively stable. Absolute prevalence increased from 27.31 million (95% UI: 24.65–30.57) in 2014 to 27.54 million (95% UI: 24.65–31.27) in 2023, corresponding to a 0.8% increase (Figure 1C). However, this did not represent a significant linear trend over time (APC −0.43%/year; 95% CI: −1.43 to 0.57; *p* = 0.348). Absolute YLDs decreased slightly from 8.16 million (95% UI: 5.70–10.84) in 2014 to 8.02 million (95% UI: 5.64–10.86) in 2023, corresponding to a 1.7% decrease (Figure 1D), also without a significant linear trend (APC −0.73%/year; 95% CI: −1.77 to 0.32; *p* = 0.147). These findings indicate that although age-standardized rates declined significantly, the absolute number of individuals living with GBD-defined spinal cord lesion-related disability did not decline significantly at the global level.

Age-stratified analyses revealed a marked divergence between working-age adults and older adults. Among individuals aged ≥70 years, absolute YLDs increased from 642,425 in 2014 to 844,877 in 2023, corresponding to a 31.5% increase. Log-linear trend analysis demonstrated a significant average annual increase of 2.57% per year from 2014 to 2023 (APC +2.57%/year; 95% CI: +1.60 to +3.55; *p* < 0.001). In contrast, YLDs among individuals aged 15–49 years decreased from 4.77 million to 4.21 million, corresponding to an 11.7% decrease and a significant annual decline of 1.87% per year (APC −1.87%/year; 95% CI: −2.98 to −0.75; *p* = 0.005). Similar age-related divergence was observed for absolute prevalence: prevalence among individuals aged ≥70 years increased by 33.6%, from 2.52 million to 3.37 million, whereas prevalence among individuals aged 15–49 years decreased by 10.4%, from 15.28 million to 13.69 million.

In sex-specific analyses among adults aged ≥70 years, both males and females showed significant increases in prevalence and YLDs from 2014 to 2023. The relative increase was slightly greater among males, with YLDs increasing by 34.7% (APC +2.84%/year; *p* < 0.001), compared with a 28.4% increase among females (APC +2.30%/year; *p* < 0.001) (Table 1).

The relative contribution of older adults to the global disability burden also increased over time. Individuals aged ≥70 years accounted for approximately 7.9% of total global YLDs in 2014 and 10.5% in 2023. Conversely, the proportion of YLDs attributable to individuals aged 15–49 years declined from approximately 58.5% to 52.5%. These findings support a shift in GBD-defined spinal cord lesion-related disability burden toward older populations.

SDI-stratified analyses demonstrated that the geriatric increase in GBD-defined spinal cord lesion-related disability was present across all development levels. Among individuals aged ≥70 years, absolute YLDs increased significantly in all five SDI quintiles, with APCs of +3.36%/year in low-SDI regions (95% CI: +2.01 to +4.73; *p* < 0.001), +1.78%/year in low-middle SDI regions (95% CI: +0.10 to +3.48; *p* = 0.040), +1.97%/year in middle-SDI regions (95% CI: +0.41 to +3.55; *p* = 0.019), +3.25%/year in high-middle SDI regions (95% CI: +1.71 to +4.81; *p* = 0.001), and +2.46%/year in high-SDI regions (95% CI: +1.74 to +3.18; *p* < 0.001). In contrast, trends among individuals aged 15–49 years varied substantially by SDI level. YLDs increased significantly in low-SDI regions (APC +2.11%/year; 95% CI: +1.67 to +2.55; *p* < 0.001), showed no statistically significant change in low-middle SDI regions (APC +0.27%/year; 95% CI: −0.39 to +0.94; *p* = 0.378), and declined significantly in middle-SDI regions (APC −1.26%/year; 95% CI: −2.06 to −0.45; *p* = 0.007), high-middle SDI regions (APC −2.65%/year; 95% CI: −3.91 to −1.38; *p* = 0.001), and high-SDI regions (APC −3.46%/year; 95% CI: −4.88 to −2.03; *p* < 0.001).

Anatomical-level analyses showed that cervical-level lesions accounted for the majority of global GBD-defined spinal cord lesion-related disability burden. Globally, the cervical ratio increased from 62.8% in 2014 to 63.5% in 2023. A persistent SDI gradient in anatomical burden distribution was observed, with the cervical ratio increasing from 55.4% in low-SDI regions to 57.9% in low-middle SDI regions, 60.0% in middle-SDI regions, 61.9% in high-middle SDI regions, and 68.3% in high-SDI regions in 2023. This pattern indicates that the proportion of disability burden attributable to cervical-level lesions was highest in more socio-demographically developed regions. The global, anatomical, and age-specific temporal trends are summarized in Table 2, while SDI-stratified YLD trends and cervical ratio patterns are summarized in Table 3.

## 4. Discussion

### 4.1. Global Trends in GBD-Defined Spinal Cord Lesion Burden

This global analysis from 2014 to 2023 highlights important shifts in the modeled epidemiology and disability burden of GBD-defined spinal cord lesions, with particular emphasis on older adults, socio-demographic development, and anatomical level. Understanding evolving epidemiological patterns of GBD-defined spinal cord lesions is essential for informing clinical practice and guiding health policy. Our findings suggest that the most consistent change was an increasing disability burden among older adults, while trends among working-age adults varied substantially by SDI level.

Our findings are broadly consistent with previous GBD-based studies showing that spinal cord injury and related spinal lesion categories remain important contributors to global disability burden, particularly among older adults. Prior global analyses have reported that age-standardized rates and absolute burden may move in different directions over time, reflecting the combined influence of demographic aging, population growth, changing injury patterns, survival, and disability duration. The present study extends this literature by focusing on the most recent 2014–2023 period using GBD 2023 estimates and by emphasizing the divergence between working-age adults and adults aged ≥70 years. Unlike broader analyses covering longer time horizons or wider injury categories, the present study specifically evaluates the modeled burden of the two GBD-defined spinal cord lesion categories and highlights their age-, SDI-, and anatomical-level patterns.

The principal finding of this study is not a uniform global increase in burden, but rather a divergence between declining age-standardized rates, relatively stable absolute global burden, and increasing disability burden among adults aged ≥70 years. Although the absolute burden remained higher among individuals aged 15–49 years, adults aged ≥70 years showed significant increases in both absolute prevalence and YLDs over the study period. This pattern may be consistent with global demographic aging and the increased vulnerability of older individuals to low-energy trauma, particularly falls; however, improved survival, changing trauma patterns, and fall-related mechanisms were not directly assessed in the present analysis and should therefore be interpreted as hypothesis-generating explanations rather than inferred causes [1,9]. This age-related shift is clinically relevant, as prior research has associated older age with higher complication rates and mortality after spinal injury [10,11,12]. As life expectancy continues to rise worldwide, older adults may contribute an increasing share of spinal injury-related disability, with potential implications for long-term care and rehabilitation systems [13]. Lu et al. [14] found that the global age-standardized prevalence of SCI increases with age, peaking around 70-year-olds. In our analysis, YLDs among adults aged ≥70 years increased significantly, whereas YLDs among individuals aged 15–49 years declined significantly, supporting an age-related redistribution of disability burden. Between 2014 and 2023, absolute YLDs in the 70+ demographic went up by 31.5% (642,425 to 844,877), while YLDs for the 15–49 age group decreased by only 11.7%. By 2023, the elderly accounted for over 10% of the total global YLD burden, a strong proportional increase from the 8.0% observed in 2014.

Notable differences were observed across Socio-demographic Index (SDI) regions. High-SDI and high-middle SDI regions consistently demonstrated the highest prevalence and YLD rates among older adults. These findings could plausibly reflect differences in population age structure, survival, diagnostic capacity, and access to rehabilitation services in higher-SDI regions. In contrast, low-SDI regions exhibited substantially lower absolute prevalence and YLD rates. However, this interpretation requires caution, as underdiagnosis, limited access to specialized care, and higher early mortality may contribute to underestimation of the true burden in low-resource settings [15]. The SDI-stratified findings add two important observations. First, the increase in YLDs among adults aged ≥70 years was observed across all SDI quintiles, suggesting that the shift toward older adults is not limited to high-resource settings. Second, trends among individuals aged 15–49 years differed by development level, with increasing YLD burden in low-SDI regions and declining burden in middle- and high-SDI regions. This divergence may reflect differences in population growth, occupational and transport-related exposures, injury prevention infrastructure, trauma care, survival, and diagnostic ascertainment. However, these mechanisms were not directly measured in the present analysis. Therefore, the SDI findings should be interpreted as population-level patterns that identify areas for further mechanism-specific research rather than as evidence of specific causal pathways.

In our analysis, neck-level lesions accounted for a greater proportion of total YLDs than below-neck lesions throughout the study period, consistent with prior literature describing the substantial disability associated with cervical-level injuries [16,17]. This finding is clinically plausible because cervical-level injuries may be associated with tetraplegia, higher dependency, and long-term disability [18]. Although absolute YLD numbers fluctuated over time, cervical-level lesions remained the dominant contributor to GBD-defined spinal cord lesion-related YLDs. This relative stability suggests that the anatomical distribution of modeled GBD-defined spinal cord lesion-related disability changed only modestly during the study period. Both neck-level and below-neck YLD estimates decreased around 2018–2020. This temporal pattern may be partly consistent with reports of reduced traumatic spinal cord injury incidence during the COVID-19 pandemic, although the present study did not directly assess pandemic-related mechanisms. McCaughey et al. [19] found a lower incidence of spinal cord injuries during the pandemic.

The stepwise increase in cervical ratio from low- to high-SDI regions was another notable SDI-related pattern. One possible explanation is that higher-SDI regions may have greater survival after severe cervical lesions and more complete diagnostic capture of long-term disability, whereas low-SDI estimates may be more affected by early mortality or underreporting. However, because GBD outputs do not include individual-level injury severity, survival, treatment, or rehabilitation data, this anatomical gradient should be interpreted cautiously as a descriptive finding.

The increasing YLD burden among older adults has potential implications for healthcare planning, particularly for rehabilitation, long-term care, and geriatric spine services. Aging societies may require greater attention to geriatric-focused rehabilitation services, long-term care infrastructure, and fall-prevention strategies. Furthermore, the predominance of cervical-level YLD burden supports the clinical importance of early specialized management and multidisciplinary rehabilitation, although treatment pathways were not directly evaluated in this study. Health systems should consider the possibility of increasing rehabilitation and long-term care needs among older adults with GBD-defined spinal cord lesions, even if age-standardized rates continue to decline [14].

### 4.2. Strengths and Limitations

The primary strength of this study lies in its use of standardized Global Burden of Disease 2023 estimates, allowing for comprehensive analyses across age groups, regions, and time. The focus on prevalence and YLD provides a clinically meaningful assessment of long-term disability burden. Several additional limitations should be considered. First, GBD estimates are model-based and depend on the availability and quality of source data, which vary substantially across countries and SDI regions. Second, the GBD Injuries by Nature categories used in this study should not be interpreted as individual-level, clinically adjudicated neurological spinal cord injury diagnoses. Third, the available GBD outputs do not provide neurological severity, ASIA Impairment Scale grade, detailed vertebral level, injury mechanism, treatment type, rehabilitation access, or long-term functional outcomes. Fourth, prevalence and YLDs reflect population-level disability burden and should not be interpreted as direct measures of acute-care demand or surgical need. Finally, explanations related to falls, trauma systems, survival, diagnostic capacity, and rehabilitation availability are plausible interpretations but were not directly tested in the present analysis This study's limitations are comparable to those of previous GBD research [20,21,22].

## 5. Conclusions

This GBD 2023 analysis suggests that although age-standardized prevalence and YLD rates associated with GBD-defined spinal cord lesions declined globally from 2014 to 2023, the absolute burden remained relatively stable and shifted toward older adults. The increase in YLDs among adults aged ≥70 years was observed across all SDI quintiles, while working-age trends differed by development level. Cervical-level lesions accounted for the majority of GBD-defined spinal cord lesion-related YLDs, with a higher cervical ratio in higher-SDI regions. These findings support the need to consider age structure, socio-demographic development, and anatomical distribution when planning rehabilitation services, long-term care capacity, and future epidemiological studies.

## Figures and Tables

**Table 1:** Sex-specific trends in GBD-defined spinal cord lesion burden among adults aged ≥70 years, 2014–2023.

Sex	Prevalence Trend	YLD Trend	Relative Pattern
Male	+36.7%; APC +3.04%/year, *p* < 0.001	+34.7%; APC +2.84%/year, *p* < 0.001	Higher relative increase
Female	+30.7%; APC +2.54%/year, *p* < 0.001	+28.4%; APC +2.30%/year, *p* < 0.001	Lower relative increase

Abbreviations: APC, annual percent change; YLD, years lived with disability.

**Table 2:** Global, anatomical, and age-specific temporal trends in GBD-defined spinal cord lesion burden, 2014–2023.

Stratification Category	Metric of Measure	2014 Estimate (95% UI)	2023 Estimate (95% UI)	10-Year Change	Trend Analysis
Global Burden	Absolute Prevalence	27.31 M (24.65–30.57)	27.54 M (24.65–31.27)	+0.8%	APC −0.43%/year; *p* = 0.348
	Absolute YLDs	8.16 M (5.70–10.84)	8.02 M (5.64–10.86)	−1.7%	APC −0.73%/year; *p* = 0.147
	Age-standardized prevalence rate	363.17 (327.87–406.32)	318.28 (284.00–363.52)	−12.4%	APC −1.97%/year; *p* = 0.002
	Age-standardized YLD rate	108.41 (75.76–144.00)	93.25 (65.44–126.19)	−14.0%	APC −2.19%/year; *p* = 0.001
Anatomical Level	Cervical-level absolute YLDs	5.13 M (3.61–6.61)	5.10 M (3.59–6.70)	−0.5%	APC −0.60%/year; *p* = 0.210
	Cervical ratio	62.8%	63.5%	+0.7 pp	+0.08 pp/year; *p* < 0.001
	**Below-Neck** absolute YLD	3.04 M (2.09–4.23)	2.93 M (2.04–4.16)	−3.7%	APC −0.96%/year; *p* = 0.084
Age Dynamics	Elderly (70+)—Absolute Prevalence	2.52 M (2.2–2.8)	3.37 M (3.0–3.8)	+33.6%	APC +2.78%/year; *p* < 0.001
	Elderly (70+)—Absolute YLD	642 k (541–752)	845 k (712–991)	+31.5%	APC +2.57%/year; *p* < 0.001
	Young Adult (15–49)—Absolute Prevalence	15.28 M (12.2–15.1)	13.69 M (10.9–13.5)	−10.4%	APC −1.70%/year; *p* = 0.007
	Young Adult (15–49)—Absolute YLD	4.77 M (3.9–5.4)	4.21 M (3.4–4.8)	−11.7%	APC −1.87%/year; *p* = 0.005

Abbreviations: APC, annual percent change; UI, uncertainty interval; YLD, years lived with disability; pp, percentage points.

**Table 3:** SDI-stratified YLD trends and cervical burden ratio, 2014–2023.

SDI Level	Cervical Ratio (2023)	≥70 YLD Change; APC	15–49 YLD Change; APC
High SDI	**68.3%**	+28.8%; APC +2.46%/yr; *p* < 0.001	−22.9%; APC −3.46%/yr; *p* < 0.001
High–Middle SDI	**61.9%**	+42.9%; APC +3.25%/yr; *p* = 0.001	−16.8%; APC −2.65%/yr; *p* = 0.001
Middle SDI	**60.0%**	+28.4%; APC +1.97%/yr; *p* = 0.019	−6.0%; APC −1.26%/yr; *p* = 0.007
Low–Middle SDI	**57.9%**	+25.7%; APC +1.78%/yr; *p* = 0.040	+6.0%; APC +0.27%/yr; *p* = 0.378
Low SDI	**55.4%**	+41.0%; APC +3.36%/yr; *p* < 0.001	+20.7%; APC +2.11%/yr; *p* < 0.001

Abbreviations: APC, annual percent change; SDI, Socio-demographic Index; YLD, years lived with disability.

## Data Availability

The data analyzed in this study are publicly available through the Global Burden of Disease Results Tool, provided by the Institute for Health Metrics and Evaluation.
